# Cancer and palliative care in COVID-19 and other challenging situations—highlights from the Uganda Cancer Institute—Palliative Care Association of Uganda 3rd Uganda Conference on Cancer and Palliative Care, 23–24 September 2021, held in Kampala, Uganda and virtually

**DOI:** 10.3332/ecancer.2021.1333

**Published:** 2021-12-13

**Authors:** Julia Downing, Nixon Niyonzima, Eddie Mwebesa, Innocent Mutyaba, Henry Ddungu, Lisa Christine Irumba, Ludoviko Zirimenya, Diana Basirika, Immacula Mbarusha, Charity Kobusingye, Margaret Happy, Alfred Jatho, Dorothy Olet Adong, Cynthia Kabagambe, Collins Mpamani, Zaitun Nalukwago, Zipporah Kyomuhangi, Joyce Zalwango, Jackson Orem, Mark Mwesiga

**Affiliations:** 1 International Children’s Palliative Care Network, Suite 1b, Whitefrairs, Lewins Mead, Bristol BS1 2NT, UK; 2 Palliative care Education and Research Consortium, PO Box 6245, Kyadondo Block 262, Plot 9, Kibuye, Makindye, Kampala, Uganda; 3 Makerere University, University Rd, Kampala, Uganda; 4 Uganda Cancer Institute, Upper Mulago Hill Rd, Kampala, Uganda; 5 Hospice Africa Uganda, Mobutu Road, Kampala, Uganda; 6 Palliative Care Association of Uganda, Block 383, Plot 8804, Kitende, Entebbe Road, Uganda; 7 MRC/UVRI and LSHTM Uganda Research Unit, Plot 51-59, Nakiwogo Road, Entebbe, Uganda

**Keywords:** cancer care, palliative care, Uganda, COVID-19, pandemic, policy, integration, education, research, paediatrics

## Abstract

The 3rd Uganda Conference on Cancer and Palliative Care was held in September 2021 with the theme: cancer and palliative care in COVID-19 and other challenging situations. It was hosted by the Uganda Cancer Institute and the Palliative Care Association of Uganda (UCI-PCAU). The conference was held virtually, with a mix of pre-recorded sessions, plenary sessions being broadcast live on television (TV) by the Uganda Broadcasting Corporation TV, live speakers at the studio and others presenting in real time via Zoom. The conference brought together >350 participants who participated on Zoom, along with those attending in person at the studio and those watching the plenary sessions on TV. At the heart of this joint UCI-PCAU conference was the commitment to not only continue but to improve the provision of cancer care and palliative care within Uganda. Key themes from the conference included: the importance of Universal Health Coverage; the impact of COVID-19 on the provision of cancer and palliative care; that both cancer care and palliative care are available in Uganda; education for all; the importance of working together to provide care and overcome challenges, e.g. through technology; the resilience shown by those working in cancer and palliative care; the grief experienced by so many people who have lost loved ones during the pandemic; the importance of good health seeking behaviour – prevention is better than cure; the challenge of funding; the need for health care equity for marginalised and vulnerable populations and finally we can’t wait for the world to stop COVID-19 – COVID-19 is here to stay – we need to find solutions. The last few years have seen significant challenges due to the COVID-19 pandemic; however, despite this, cancer and palliative care service provision has continued. This conference, whilst unique and very different from previous conferences, was a great opportunity to share not only amongst each other, but also to share key messages with the public through the live broadcasting of the plenary sessions of the conference.

## Introduction

Being prepared and responding well to a global pandemic is essential in order to not only manage the pandemic, but ensure that existing health services, such as cancer care and palliative care, can continue throughout the pandemic. Since the global COVID-19 pandemic began in early 2020, much of the world has been in and out of lockdown, with health services stretched to the limit, and restrictions placed on movement and care provision, that have been previously unheard of. Uganda is no exception to this, with severe lockdowns which restricted the movement, not only of health and social care professionals trying to provide care, but also of patients and their families, trying to access care. Restrictions on meeting have impacted on care provision and on education and the running of events such as conferences. Thus, this 3rd Uganda Conference on Cancer and Palliative Care run jointly by the Uganda Cancer Institute (UCI) and the Palliative Care Association of Uganda (PCAU) was breaking new ground. This was a virtual conference, with a mix of pre-recorded sessions, plenary sessions being broadcast live on television (TV) by the Uganda Broadcasting Corporation (UBC) TV, live speakers at the studio and others presenting in real time via Zoom. Delegates had the opportunity to share about their adaptability, resilience and use of technology to ensure continuity of care in the pandemic, how they reached out to the most vulnerable with messages of health and hope and how resilient caregivers, despite their exhaustion and the strains under which they have been working, sustained cancer and palliative care services. At the heart of this joint UCI-PCAU conference was the commitment to not only continue but to improve the provision of cancer care and palliative care within Uganda, with a desire to share and learn from each other, and to learn lessons that can be applied in other challenging situations, or future pandemics. Dr Jackson Orem, Executive Director of the UCI, encouraged participants that ‘this 3rd Uganda conference on cancer and palliative care presents an opportunity for Uganda to inspire Africa and the World at large with a perspective of hope in cancer prevention, research, treatment and palliative care. We are hopeful that you all find this conference rewarding, stimulating, and meaningful for the betterment of us all*’*.

The conference was held on 23–24 September 2021, 28 years after the first palliative care service was started in Uganda – a birthday worth recognising, with several of those involved from the start in attendance. PCAU is the National Association for palliative care providers in Uganda and was established in 1999 and registered as a non-governmental organisation in 2003 to support and promote the development of palliative care in Uganda. PCAUs mission is to accelerate the integration of palliative care into the Uganda health care system through (a) capacity building, (b) advocacy and awareness creation, (c) research and information and (d) governance and resources mobilisation, in order to increase access to culturally appropriate palliative care in Uganda in collaboration with partners [1]. PCAU works in partnership with the Ministry of Health (MoH), other government agencies, stakeholders, civil society and individuals, for the scale-up of palliative care services in Uganda and has 24 member organisations and 1,200 individual members [2].

UCI is a regional centre of excellence and has provided care to individuals from within Uganda, and across East Africa, conducting ground-breaking research on cancer in Africa and globally. Its functions include but are not limited to: ‘(a) developing policy on the prevention, diagnosis and treatment for cancers and on the care for patients with cancer and cancer related diseases; (b) undertaking and coordinating the prevention and treatment of cancers in Uganda; (c) providing comprehensive medical care services to patients affected with cancer and cancer related diseases; (d) providing palliative care and rehabilitation services to patients with cancer; (e) overseeing the management of cancer and cancer related services in public and private health centres; and (f) conducting or coordinating cancer related research activities in and outside Uganda’ [1, 3].

UCI [4] attends to an average of 200 patients a day. In the past 10 years, it has changed from a place where you were ‘sent to die’ to a centre of excellence in research and clinical care, and is amongst the best cancer centres in the region.

Cancer is one of the leading causes of morbidity globally and an important public health concern, with 17 million new cases annually in 2018, and 9.6 million deaths [5]. Globally rates are expected to increase to 21.6 million by 2030 [6]. Uganda is also seen as one of the leading countries in sub-Saharan Africa for the provision of palliative care [7]. Prior to the pandemic, there had been key developments in cancer and palliative care with the World Health Assembly (WHA) [6, 8] passing resolutions which are critical in guiding us into the future for cancer and palliative care service provision, the sustainable development goals, in particular that of goal 3: good health and wellbeing [9], the Declaration of Astana on Primary Health Care [10] and Universal Health Coverage (UHC) [11]. However, all of these have been superseded by the COVID-19 pandemic. As of the 26 September 2021, there had been 231.7 million confirmed cases of COVID-19 globally with 4.7 million deaths [12]. *‘*The unprecedented overload of the healthcare system due to the pandemic presented hospitals around the world with high demands on structural and operational capacities placed many medical facilities on the brink of collapse. As a result, the high influx of severe patients required hospitals to expand intensive care units and bed capacities using other units including operating rooms and close non-essential services*’* [12]. Alongside this, countries including Uganda have had to look at how they can contain and control the epidemic, initiating national lockdowns, restricting travel both within and outside of the country, requiring self-isolation of those in contact with people with COVID-19 and restricting access to some health care services. Therefore, alongside the challenges of not having access to personal protective equipment (PPE) and the increased cost of living, health and social care professionals have found it hard getting to work, providing care, caring for their families and staying safe. It is within this context that cancer and palliative care services have been provided during the pandemic, and it is within this context that participants gathered virtually at the 3rd Uganda Conference on Cancer and Palliative Care.

## Opening of the conference

In the opening plenary of the conference, Dr Tedros Adhanom, Director General of the World Health Organization (WHO), Dr Charles Olaro, Director Curative Health Services on behalf of the Hon. Minister for Health and the Rt. Hon. General Moses Ali, First Deputy Prime Minister and Deputy Leader of Government Business in Parliament, on behalf of the Prime Minister of the Republic of Uganda, all spoke about the resilience and perseverance seen throughout the pandemic. Dr Tedros, in a pre-recorded speech, recognised the impact of the pandemic on both cancer and palliative care services throughout the world. He congratulated Uganda for innovative and creative ways of maintaining services despite the pandemic. He noted that there will be new technical documents published in the coming weeks for palliative care and highlighted the importance of why UHC must remain our top priority built on resilient health systems that can continue to deliver essential health services for cancer care including palliative care, even in the most serious crisis. Dr Olaro recognised the importance of the cancer control continuum emphasising the need to focus on prevention, early detection, diagnosis, treatment and palliative care [13]. He emphasised the commitment of the MoH for cancer and palliative care noting that the Ministry has created a division of palliative care in the Department of Clinical Services headed by Dr Jackson Amone, and that they have created positions in Kiruddu and Kawempe Hospitals, as well as creating positions throughout the health system for specialists in palliative care. The government works in partnership with both palliative care and cancer care and has seen the UCI develop as a centre of excellence in care, research and training. He stated, on behalf of the Minister of Health that the Government of Uganda is committed to increasing access to quality cancer and palliative care for all its citizens throughout the country. The Rt. Hon. General Ali also emphasised the government’s commitment to expanding access to cancer and palliative care through prioritising access to cancer services in the National Development Plan III [14] and the establishment of regional cancer centres in four regions in Uganda – Arua, Mbarara, Mbale and Gulu, reducing the need for cancer patients to be referred abroad, improving gradually on the budgetary allocation for cancer services, covering the cost of essential palliative care medicines. He referred to the WHA resolutions on cancer [6] and palliative care [8] where countries were called upon to invest in and integrate services, and that the Government is committed to fulfilling these resolutions. In order to do this, we need to develop early diagnosis programmes, develop partnerships, train health professionals and strengthen networks of direct stakeholders. On officially opening the conference, he wished all the presenters, clinicians, researchers and policy makers attending a fruitful and successful conference.

## Conference summary

**Figure 1.**
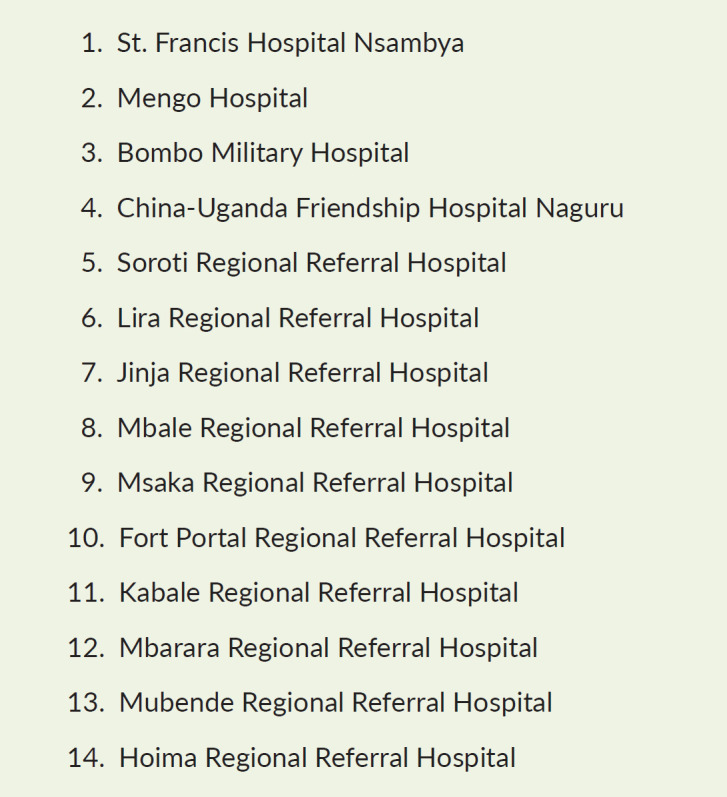
Satellite facilities across Uganda.

The conference, held virtually and in the UBC studios, brought together >350 delegates who participated on Zoom, along with those speakers attending in person and members of the public who were able to watch the main conference plenary sessions live on TV via UBC. This was the first time that the joint conference had gone out live on TV, thus increasing the impact as the public, along with other health professionals, policy makers, etc., were able to join via TV. Important to PCAU’s focus area of increasing awareness on palliative care, but also meaning that it was hard to estimate reach, although clearly this conference has had a much broader reach than any previous face-to-face meeting. Bearing in mind the challenges that some people can experience with regard to the Internet and technology, satellite facilities were set up in 14 health facilities (Figure 1), thus enabling participants to come together locally and join the conference together and once again increasing reach. The conference brought together a wide range of clinicians, academics, human rights advocates, lawyers, clergy, researchers, social workers, policy makers, MoH officials and donors, representing over 100 organisations, to share lessons and look at how services had adapted in order to survive. All of the sessions were interpreted into sign language which was also visible on UBC. The conference was organised into four main tracks as follows:

1. Innovation and creativity
2. Providing care to vulnerable populations
3. Sustainability of cancer and palliative care services
4. Resilience and caring for the caregivers

The scientific programme included 14 plenary presentations across 4 plenary sessions, 33 oral breakout presentations, 2 workshops and 21 poster presentations. Presentations were given across the continuum of care, from prevention through to end-of-life care, across the age span, and whilst the main focus was on cancer care, the provision of palliative care for other conditions was also addressed. During the opening ceremony, both UCI and PCAU gave some recognition awards. The UCI recognition award went to Mr Matovu Wasswa who is a swimming coach who lost his wife to cancer. He noticed how many children at UCI and their families struggled for food whilst receiving treatment and so he has mobilised friends and family to give food supplements to the children’s ward – something he has continued as much as possible throughout the pandemic. The UCI institutional award went to Development Finance Company of Uganda Bank Ltd. (DFCU) who have supported awareness campaigns but also paid for a shelter for patients who are waiting for medication, amongst other things. The PCAU individual award went to Sr Catherine Nakasita, a Senior Assistant Nursing Officer in Kitagata Hospital, who developed a palliative care unit, and ensured that during the pandemic, patients continued to receive care at home. The PCAU Institutional award went to Joy Hospice Mbale. Joy Hospice was the first place to record a known COVID-19 death in Uganda, which was very distressing for them and the other patients at the hospice and meant that they all had to go into isolation. They have continued to provide care throughout the pandemic and have shown resilience, determination and commitment. Both UCI and PCAU said how hard it had been to choose the winners for the awards as so many people have been dedicated, innovative and committed throughout the pandemic and they thanked everyone for their ongoing work.

### Key conference themes

The key themes identified from the conference were as follows:

1. The importance of UHC and having appropriate policies in place
2. The impact of COVID-19 on the provision of cancer and palliative care services
3. That both cancer care and palliative care are available in Uganda
4. Education for all, including strengthening nursing and midwifery
5. The importance of working together to provide care and overcome challenges, e.g. through technology
6. The resilience shown by those working in cancer and palliative care
7. The grief experienced by so many people who have lost loved ones during the pandemic – no family has not been affected by COVID-19
8. The importance of good health seeking behaviour – prevention is better than cure
9. The challenge of funding
10. The need for health care equity for marginalised and vulnerable populations
11. We can’t wait for the world to stop COVID-19 – COVID-19 is here to stay – we need to find solutions.

### The importance of UHC and having appropriate policies in place

From the start of the conference with the opening words from Dr Tedros, the importance of UHC was emphasised. The WHO [15] states that ‘UHC means that all individuals and communities receive the health services they need without suffering financial hardship. It includes the full spectrum of essential, quality health services, from health promotion to prevention, treatment, rehabilitation, and palliative care across the life course*’*. The COVID-19 pandemic has thrown into the spotlight where there are weaknesses in our health systems, the essential nature of UHC and the importance of providing care across the life course, from health promotion through to palliative care.

In order to achieve this, we must have the appropriate policies in place. During the pandemic, there has been a plethora of COVID-19 related standard operating procedures (SOPs) developed and implemented, along with policies and procedures at all levels. However, other policies, such as the palliative care policy for Uganda are still not passed, and yet there is an urgent need for this for the implementation of UHC. The WHA resolutions on cancer [6] and palliative care [8] both highlight the need for policies to guide practice, but also for ensuring education is available for all. For example, the WHA resolution on palliative care [8] states that governments need *‘*to develop, strengthen and implement, where appropriate, palliative care policies to support the comprehensive strengthening of health systems to integrate evidence-based, cost-effective and equitable palliative care services in the continuum of care, across all levels, with emphasis on primary care, community and home-based care, and universal coverage schemes*’*. Likewise, the Cancer Resolution recommends reducing risk factors for cancer though policies and programmes with the WHO committing to supporting countries to strengthen the policy environment [6]. Having the appropriate policies will also help in the implementation of UHC, and the Sustainable Development Goals. [9, 11] Whilst appreciating all that the Government is doing for palliative care, several of the speakers also recommended that the government should ensure that the palliative care policy is finalised. Dr Charles Ayume, Chair of the Committee of Health in the Parliament of Uganda, in his paper on the second day of the conference pledged to fast track the Palliative Care Policy and get it approved and finalised, along with being committed to finance palliative and cancer care. The policy is also key in addressing issues of health systems strengthening and financing for cancer and palliative care.

Key to the provision of UHC, cancer and palliative care is the pain management, a topic explored through a workshop on integrating pain management into routine service care delivery at hospitals in Uganda. A range of presenters explored the importance of pain assessment and management, including Dr Fred Sebisubi, the former Assistant Commissioner, Department of Pharmaceuticals and Natural Medicines, who represented Dr Neville Oteba, the current Commissioner, Department of Pharmaceuticals and Natural Medicines at the MoH, Rinty Kintu from the American Cancer Society, Dr Emmanuel Higenyi from Joint Medical Stores, Kenneth Mwehonge from Coalition for Health Promotion and Social Development (HEPS) Uganda, Rosemary Canfua from Treat the Pain and Joyce Zalwango from PCAU. During the workshop, there was recognition that pain is distressing and if we can manage pain, we can go a long way in improving quality of life. The experience of increasing access to pain assessment and management in Uganda through the Pain Free Hospital Initiatives Programme was shared, along with lessons from other countries within the region. The importance of all aspects of the supply chain was also stressed, and sadly this had become unstable during COVID-19. The importance of the control of medicines was also discussed but it was highlighted that this should not hinder access to pain management. The government of Uganda is supporting hospitals to ensure that all facilities integrate pain management into routine care and the consumption of morphine has increased with 226 accredited facilities to prescribe and dispense oral morphine in 107 districts of Uganda, although it is still low based on international standards.

### The impact of COVID-19 on the provision of cancer and palliative care services

The impact of the pandemic on the provision of cancer and palliative care services has been great, and many of the abstract presenters talked about how COVID-19 had impacted on the provision of their services and therefore patient outcomes with Mark Mwesiga from PCAU saying that although COVID-19 impacted negatively on access to palliative care in Uganda, we quickly restructured the services to respond to the demand for palliative care across the country. Dr Emmanuel Luyirika from the African Palliative Care Association (APCA) discussed the status of palliative care within the region before COVID-19, and currently. Over the years, Uganda has been seen as a leader in palliative care within the region, with work from 2017 demonstrating that Uganda came on top within the region for the number of services, with 5.87 palliative care services per million inhabitants [7]. When the Global Atlas on Palliative Care was published in 2020, during the epidemic [16], Uganda was no longer on top with Malawi and Eswatini having integrated palliative care. APCA along with Kings College London carried out a survey across Africa to see how prepared services were to respond to a pandemic [17] with issues being highlighted such as water, soap, disinfectants and PPE, with security of staff being key. Since the pandemic started, many palliative care programmes have been closed or have reduced services due to the financial impact of the pandemic. Fewer patients have also been seen due to lockdowns, challenges with PPE, transport, etc., yet at the same time the need for services was increasing. Services had to also respond to a large amount of mis-information about the virus, vaccinations, etc., adding an extra challenge. Recommendations were made to continue to increase and ring-fence funding for cancer and palliative care even in an epidemic.

Dr Joyce Balagadde talked about the impact of the pandemic on childhood cancers in Uganda. Globally it has been recognised that childhood cancer has been affected by the pandemic with >30,000 children impacted globally [18], due to the restrictions, etc., that individual governments and health systems have put in place – these children have become a casualty of the pandemic. Some of the challenges in Uganda have been that children have found it difficult to access cancer treatment due to national lockdown, the grounding of public transportation, families and guardians who have lost their livelihoods or have died. The impact of the pandemic on health professionals has also been great with >50% of the team at UCI being hit by COVID-19, and services being suspended. Therefore, COVID-19 threatens to reverse the gain that Uganda has made on childhood cancer – UCI saw 25% of the 1,000 children that they expected, and the majority of those were in the advanced stages of disease with poor adherence to treatment exacerbated by lockdown restrictions. Dr Joyce recommended that during challenging situations such as a pandemic, we find ways to get children into care and prevent them from abandoning treatment. She finished by saying: *‘*Let us find services and solutions to care for our children making sure that they live with dignity despite COVID-19*’*.

Other presenters shared lessons learnt from across the country including Mbale Regional Referral Hospital, Mobile Hospice Mbarara, UCI, Little Hospice Hoima and Kawempe Home Care. Lisa Irumba from PCAU also shared how they had established SOPs for the continuity of palliative care services throughout the pandemic, working hand in hand with partners and in collaboration with the MoH. These SOPs built confidence amongst palliative care programmes and helped to ensure safety for both patients and caregivers whilst continuing to offer essential services.

### That both cancer care and palliative care are available in Uganda

Throughout the conference, there was an emphasis on the fact that cancer care is available in Uganda – that care is provided through the UCI and that regional cancer hubs are being developed. Professor Jackson Orem, Executive Director of UCI, sets the scene at the start of the conference highlighting the work of the UCI, and presenters throughout the conference spoke about the care being provided by UCI and its recognition as a Centre of Excellence in East Africa. Dr Abrahams Omoding stressed that no longer do people need to think that going abroad is the only source of hope for cancer treatment, as Uganda has built the infrastructure and has the team available to provide care. Dr Victoria Walusansa, the Deputy Executive Director of UCI, shared how four regional cancer centres are being established in a phased manner to decongest UCI in Kampala – for example, Mbarara Regional Cancer Centre is operational, seeing an average of 1,000 cancer patients a year.

Mr Mark Mwesiga from PCAU and Dr Agasha Birungi from Hospice Africa Uganda both stressed that 107 districts have access to palliative care services [2], with health facilities having been accredited to provide such services, and health professionals trained. It was also stressed that palliative care is not just for those at the end of life, but that palliative care, given earlier in the course of the illness, can improve quality of life and enable individuals to go back to work, etc.

These messages were highlighted both in terms of cancer care and palliative care throughout the plenary sessions which were being broadcast live on UBC as they are key messages for the public. Too often there is a view that such services are not available in Uganda, and we need to change that view and ensure that all Ugandans have access to both cancer and palliative care services when they need it.

Throughout the conference, a range of cancer and palliative care services were discussed, giving evidence of the local provision of services, for example, palliative care for those with end-stage renal disease, radiotherapy services, the care of individuals with Kaposi sarcoma.

### Education for all, including strengthening nursing and midwifery

Throughout the presentations, there was an emphasis on the need for education at all levels of care, in order to ensure that signs of cancer are picked up in the Health Centres, with appropriate referrals to the District, Regional and National Referral Hospitals. The WHA resolution on cancer [6] recommends the optimising of existing human resources and anticipating future requirements for cancer prevention and control which includes education and training, Likewise the WHA resolution on palliative care [8] also stressed the importance of including ‘palliative care as an integral component of the ongoing education and training offered to care providers, in accordance with their roles and responsibilities, according to the following principles:

- Basic training and continuing education on palliative care
- Intermediate training for all routinely working with patients with life-threatening illnesses
- Specialist palliative care training’.

The importance of education and training was highlighted by Dr Charles Olaro at the opening of the conference, and was a theme addressed throughout the conference. In the panel discussion on day 1, it was emphasised that every health worker needs knowledge on cancer and palliative care, as patients will be seen throughout the health system and this can only be done through education and training. Rinty Kintu also emphasised that in order to strengthen access to pain relief medicines in Africa, we need to train many health workers and educate the public in order to reduce misconceptions about the use of morphine and other analgesics. Dr Nixon Niyonzima stressed the need for multi-disciplinary education, and the need for both specialist and generalist education. He discussed some of the challenges to this including an inadequate infrastructure, a lack of specialist training programmes, a lack of trainees, insufficient funding and a lack of enabling policies. However, he also noted that there are many opportunities to address these challenges through establishment of local training programmes and strengthening existing ones, increased funding for training and research and creating enabling policies, thus improving working conditions. Aggrey Kibenge stressed the importance of this to address health equity for marginalised people with education and training key, Florence Nalutaaya described an education programme on children’s palliative care delivered during the pandemic and Rays of Hope Hospice Jinja described how they improved palliative care accessibility through training health workers in the rural areas. Providing education throughout the pandemic has been a challenge but the team from the Institute of Hospice and Palliative Care in Africa (IHPCA) showed that with proper planning, preparations and strict adherence of SOPs, face-to-face training could be successfully conducted without spreading COVID-19 as they delivered the face-to-face components of their bachelors and masters programmes.

The importance of educating nurses was also highlighted by Sr Amuge Beatrice, Commissioner incharge of Nursing at the MoH, who discussed the role of nurses in cancer and palliative care during the pandemic, linking in with the recently approved Global Strategic Directions for Nursing and Midwifery [19] which focuses on education, availability of health workers, leadership and service delivery. She stressed the role that nurses have played during the pandemic in cancer and palliative care and will continue to be, thus it is essential that they are adequately prepared and supported.

### The importance of working together to provide care and overcome challenges, e.g., through technology

Throughout the presentations, it was very clear that we cannot work in isolation, or as individuals, but that it has been as we have been working together to overcome the COVID-19 pandemic that we have been having success. In their opening, both Dr Olaro and Rt. Hon. General Ali talked about the fact that Uganda has only got to where it is through working together as partners, to sharing the load, and through enabling others. Dr Orem stressed the fact that collaboration is paramount in national cancer control, and that our combined resources and solidarity are necessary for fighting cancer, including cancer control research and policy. Cyndy Searfoss shared how access to cancer and palliative care can be strengthened through collaboration, both local, national and international. Through collaboration, we can address challenges and we are stronger when we work together. Indeed, the Rt. Hon. General Ali reiterated that the Government is committed to developing partnerships to lead to improved quality of service provision. This was a sentiment echoed in many of the presentations.

One key area where collaboration had been key was in the development and utilisation of IT, e.g., through the utilisation of databases and digital technology – an area that has been brought to the forefront of health service delivery and education through the pandemic. With countries, such as Uganda, coming to rely on different digital technology to enable them to continue to provide care and support. Elizabeth Nabirye from the Palliative Care Education and Research Consortium (PcERC) described the lessons learnt from a study looking at health professionals’ perceptions of the use of digital technology in palliative care from Uganda, Zimbabwe and Nigeria. Results showed that they supported the use of digital technology in palliative care, identifying potential benefits but also challenges. Eve Namisango from the APCA also discussed the development and evaluation of a mobile phone-based intervention to facilitate continuity of care, symptom monitoring and self-management in patients with advanced cancer during the pandemic, in a refugee settlement in North-western Uganda. This work is still under way but initial results suggest that digital technology approaches are acceptable in augmenting palliative and cancer care in Uganda despite network and other challenges.

One of the key action points from the workshop on Improving National data on palliative care services in Uganda, was the need for a proper data management system for palliative care across Uganda. The workshop explored work being undertaken by the MoH and PCAU to improve national data on palliative care services through an mHealth project, with four pilot facilities having been scaled up so that there are now 20 health facilities involved in the project. Importantly the data collection tool piloted has been integrated into the Health Management Information System. This collaborative work is work in progress and the MoH will continue to take the lead supported by PCAU. There is a need to refine key indicators for palliative care and work has been ongoing led by the WHO with regard to international indicators, which were launched in October 2021.

### Resilience and caring for the caregivers

Both Dr Olaro and Hon. General Ali spoke about the resilience of the health professional during this time of COVID-19 with the hope that this will continue. This was emphasised by Dr Anne Merriman whilst speaking on compassionate care during life-threatening epidemics in Africa as she discussed the role of compassion and how our attitudes can make all the difference in the care that we provide. Supporting front-line care workers has been key to all cancer and palliative care organisations throughout the pandemic. Rays of Hope Hospice Jinja gave an example of how they tried to maintain both physical and mental health of frontline workers during the pandemic. Throughout the pandemic, PCAU was also looking at how they can support frontline palliative care providers. One way, presented at the conference by Joyce Zalwango was to provide psychosocial support in order to enhance their resilience and enable them to maintain the momentum of care for all patients in need of palliative care at this time. This also included a series of webinars to discuss selected topics, establishing a toll-free line for psychosocial support and developing a module for the MoH on continuity of palliative care. Resilience of health professionals was not the only concern with PCAU also sharing their work on supporting children who are heading families with adults or children receiving palliative care to be resilient during the pandemic, which demonstrated the importance of strong social support systems.

### The grief experienced by so many people who have lost loved ones during the pandemic – no family has not been affected by COVID-19

There was an acknowledgement from the start of the conference that the pandemic has been difficult for most people, that many of us have loved ones who are sick or who have died from COVID-19, and that every family will have been impacted on one way or another. There was a recognition of all those who have died due to the pandemic, with a special tribute being made to Tom Duku, who lost his life to COVID-19 and was the Chair of the Board of Hospice Africa Uganda and formerly a board member of PCAU.

### The importance of good health seeking behaviour – prevention is better than cure

Whilst there was recognition of the services available for treating cancer, it was also stressed that prevention is better than cure. If Uganda is to have a strong National Cancer Control Plan, then prevention should be a key part of this [13] and we all need to be taking as many opportunities as we can to encourage people to go forward for screening, to change their lifestyles, etc. Rose Kiwanuka described how they had integrated health promotion in palliative care within Lweza Community Health Programme and promoted health living, encouraging health seeking behaviour and minimising the occurrence of serious illness, thus impacting on patients’ quality of life.

### The challenge of funding

Funding is always raised as an issue when looking at the sustainability of cancer and palliative care services in Uganda. Never more is that the case than now during the COVID-19 pandemic. With limited resources having been redirected, many services have had to reduce their capacity due to reallocation of funding due to the funds being diverted elsewhere in relation to the pandemic. Different options for financing services were explored by Dr Charles Ayume as he discussed health systems strengthening and financing for cancer and palliative care. He stressed the importance of seeing how cancer and palliative care fit into the national strategy for disease preparedness. Funding should not be reliant on one or two individuals, but is everyone’s business, and this was important to stress, particularly thinking of those whose funding is about to be cut.

### The need for health care equity for marginalised and vulnerable populations

This is such an important topic and one that needs to be addressed, and indeed it was raised as a concern from the start of the conference. With many marginalised and vulnerable populations living in Uganda, Aggrey Kibenge discussed the importance of health care equity within the country. We know that fatality is unevenly distributed due to the challenges of accessing health care, which has been increased through the pandemic and the resulting lockdown and social economic issues, with rising unemployment pushing more households into poverty and soaring costs for medical care. Whilst the government has responded, the battle of health care equity is not likely to end any time soon, and we need to work together for every Ugandan as health care is not only an essential service, but a basic human right.

Professor Wilson Acuda from IHPCA outlined the challenges faced by vulnerable populations with life-threatening illnesses in accessing palliative care, highlighting the need for research that can inform the development of appropriate policies and interventions for vulnerable populations. Likewise, a review of cultural and religious beliefs and traditions affecting access to cancer and palliative care in Uganda underlined the significant knowledge gaps in this area. It identified the need for education to demystify myths and false beliefs as well as a multi-stakeholder adoption of culturally appropriate and affordable care. A study by Dr Barnabas Atwiine from the Mbarara University of Science and Technology, which won the award for the best oral presentation, also highlighted caregivers’ reasons for abandoning their children’s cancer treatment in south west Uganda – reasons cited included financial difficulty, other obligations, the child appearing cured, preference for alternative treatments, beliefs that cancer was incurable, side effects of treatment and fear that the child’s death was imminent. Another area of concern was that of the ‘girl-child’ and the team at the Rays of Hope Hospice in Jinja highlighted how the COVID-19 pandemic and the strict lock-down measures created increased insecurity for already poor families, and how they had supported 126 children during the lockdown period, whilst a team from Makerere University looked at the care of children with severe malnutrition. Continuing with the theme of vulnerable populations, Liz Nabirye from PcERC talked about the integration of palliative care into healthcare provision for South Sudanese refugees and host communities in Adjumani and Obongi districts – sharing the results of a rapid systems appraisal which demonstrated the need for palliative care service integration, with the refugee and host communities having ownership of service provision.

### We can’t wait for the world to stop COVID-19 – COVID-19 is here to stay – we need to find solutions

Sadly, this was a theme that was raised throughout the conference. COVID-19 is here to stay, we will find ways to work around it, but we need to find solutions to ensure that cancer, palliative care and other essential health services are provided despite the pandemic. Many lessons have been learnt through the COVID-19 pandemic and the conference highlighted the benefit of sharing these lessons, so that we can be prepared for future pandemics or other challenging situations.

## Conclusion

The last 2 years have seen significant challenges due to the COVID-19 pandemic. Despite this, and the limitations due to ongoing lockdowns, restrictions in meeting, etc., cancer and palliative care service provision has continued. It has not been easy, but the resilience, collaboration and commitment shown by those working in these fields in Uganda have been evident, ensuring that individuals with cancer and needing palliative care, and their families have still been able to access the care that they need. This conference, whilst unique and very different from previous conferences, was a great opportunity to share not only amongst each other, but also to share key messages with the public through the live broadcasting of the plenary sessions of the conference. Thus, this conference reached out to many more people than ever before, and it is hoped that the impact of this conference will also be greater than ever before. As the conference came to a close, a panel of Professor Charles Olweny, Chair of the Board UCI, Dr Henry Ddungu, Chair of the Board PCAU, Dr Jackson Orem, Executive Director UCI and Mr Mark Mwesiga, Country Director PCAU shared their thoughts on the conference, what we have learnt through the pandemic and the way ahead. The conference was then closed with a brief highlight of the conference and the awarding of the best oral presentation award by Professor Julia Downing, International Children’s Palliative Care Network and PcERC.

It was an exciting conference to be part of, and whilst we did not have the pleasure of meeting face to face, we did have a united experience in learning from each other, joining virtually and being part of the Ugandan cancer and palliative care community. Whilst we hope that in 2 years’ time when we have the next conference, we will be able to meet together again in person, we hope that we will also have learnt from this virtual televised conference and take forward the positives, such that not only will our learning through the pandemic impact on the way that we provide care and education, but also on the way that we learn from each other through our regular conference. As Uganda moves forward in the development of UHC, and the implementation of both the cancer and palliative care WHA resolutions [6, 8], we do so in the knowledge that we have learnt together, we have grown together, we have developed together through this pandemic and most importantly we have shown that we are resilient and committed to provide cancer and palliative care for all those in need of palliative care in Uganda, regardless of diagnosis, age or where they live.

## Conflicts of interest

The authors declare that they have no conflicts of interest.
